# Commentary on “Factors associated with diabetic foot ulcers and lower limb amputations in type 1 and type 2 diabetes supported by real‐world data from the German/Austrian DPV registry”

**DOI:** 10.1111/1753-0407.70035

**Published:** 2024-12-03

**Authors:** Mostafa Javanian, Mohammad Barary, Soheil Ebrahimpour

**Affiliations:** ^1^ Infectious Diseases and Tropical Medicine Research Center, Health Research Institute Babol University of Medical Sciences Babol Iran; ^2^ Student Research Committee, School of Medical Education and Learning Technologies Shahid Beheshti University of Medical Sciences Tehran Iran

Dear Editor,

We read with great interest the article “Factors associated with diabetic foot ulcers and lower limb amputations in type 1 and type 2 diabetes supported by real‐world data from the German/Austrian DPV registry”,^1^ published in your esteemed journal. The study provides an essential contribution to understanding the risk factors for diabetic foot ulcers (DFUs) and lower limb amputations, using real‐world data from a significant cohort of adults with type 1 (T1D) and type 2 diabetes (T2D). The authors rightly highlight that sex, body height, and complications related to diabetes are associated with an elevated risk of DFUs in both T1D and T2D. Additionally, the potential for improved glycemic control and lipid management in T1D, alongside reduced smoking and alcohol consumption in T2D, is suggested as an intervention to mitigate these risks.

We commend the authors' comprehensive investigation. However, several methodological limitations warrant further discussion and consideration.

First, while the study incorporates key laboratory factors, selecting biomarkers appears somewhat restricted. Expanding the scope to include additional inflammatory markers such as erythrocyte sedimentation rate (ESR), C‐reactive protein (CRP), neutrophil‐to‐lymphocyte ratio (NLR), platelet‐to‐lymphocyte ratio (PLR), the systemic inflammation response index (SIRI), and the systemic immune‐inflammation index (SII) would provide a more nuanced understanding of systemic inflammation's role in the development of DFUs and subsequent amputations.^2^, ^3^ These markers have been shown to correlate with adverse outcomes in diabetic complications, yet they were not accounted for in this study. Including them would enhance the predictive model and potentially allow for more targeted interventions. Relevant comorbidities were either underexplored or omitted altogether. For example, cerebrovascular disease, malignancies, and psychological factors—each of which can significantly impact wound healing and overall morbidity—were not sufficiently addressed. A deeper exploration of these comorbidities could offer valuable insights into multifactorial risks associated with DFUs and amputations.

Third, a critical factor overlooked in the study is the duration of DFUs and their correlation with the likelihood of amputation. Wound chronicity is a well‐established risk for poor outcomes, and incorporating this variable into the analysis would improve the understanding of which ulcers are most likely to lead to amputation. Additionally, the study could benefit from a more detailed classification of wounds based on their anatomical location (e.g., under the metatarsal heads, heel, or malleoli). Certain anatomical regions are more prone to complications, and this granularity could improve the precision of risk stratification.

Lastly, the role of foot deformities, particularly Charcot foot, in increasing the risk for DFUs and amputations was not sufficiently addressed. Due to its severe structural changes, Charcot's foot is a critical risk factor for ulcer formation and subsequent infection, and its omission limits the study's scope. Including such deformities would provide a more comprehensive picture of the structural risk factors involved.

In conclusion, while the article offers valuable insights into DFUs and amputations among patients with diabetes, addressing these gaps could significantly enhance the robustness of the findings and provide more practical applications for clinical management. We hope the authors will consider these suggestions and that they may serve as avenues for further research in this critical area.

## AUTHOR CONTRIBUTIONS

**Mostafa Javanian:** Conceptualization, Methodology; **Mohammad Barary:** Writing – original draft, Writing – review & editing; **Soheil Ebrahimpour:** Investigation, Supervision, Writing – original draft.

## FUNDING INFORMATION

None.

## CONFLICT OF INTEREST STATEMENT

The authors declare no conflicts of interest.

## Data Availability

Data sharing is not applicable to this article as no new data were created or analyzed in this study.
